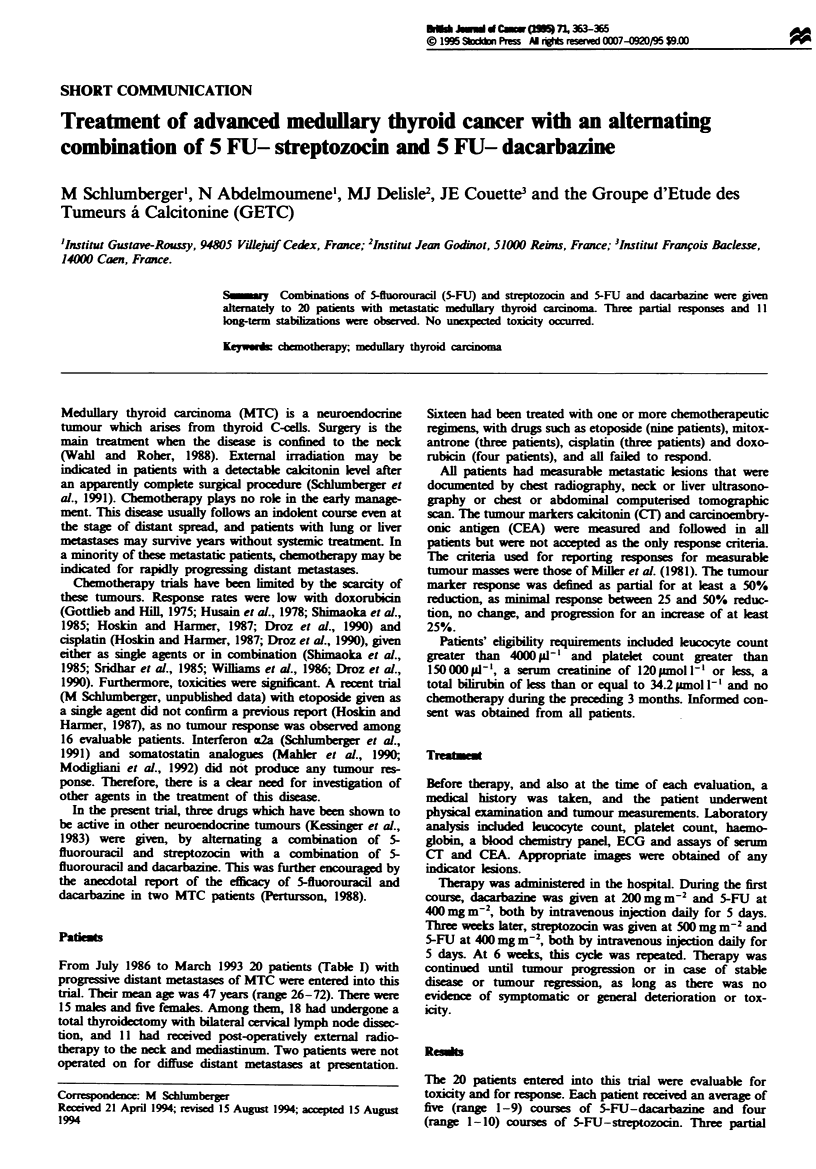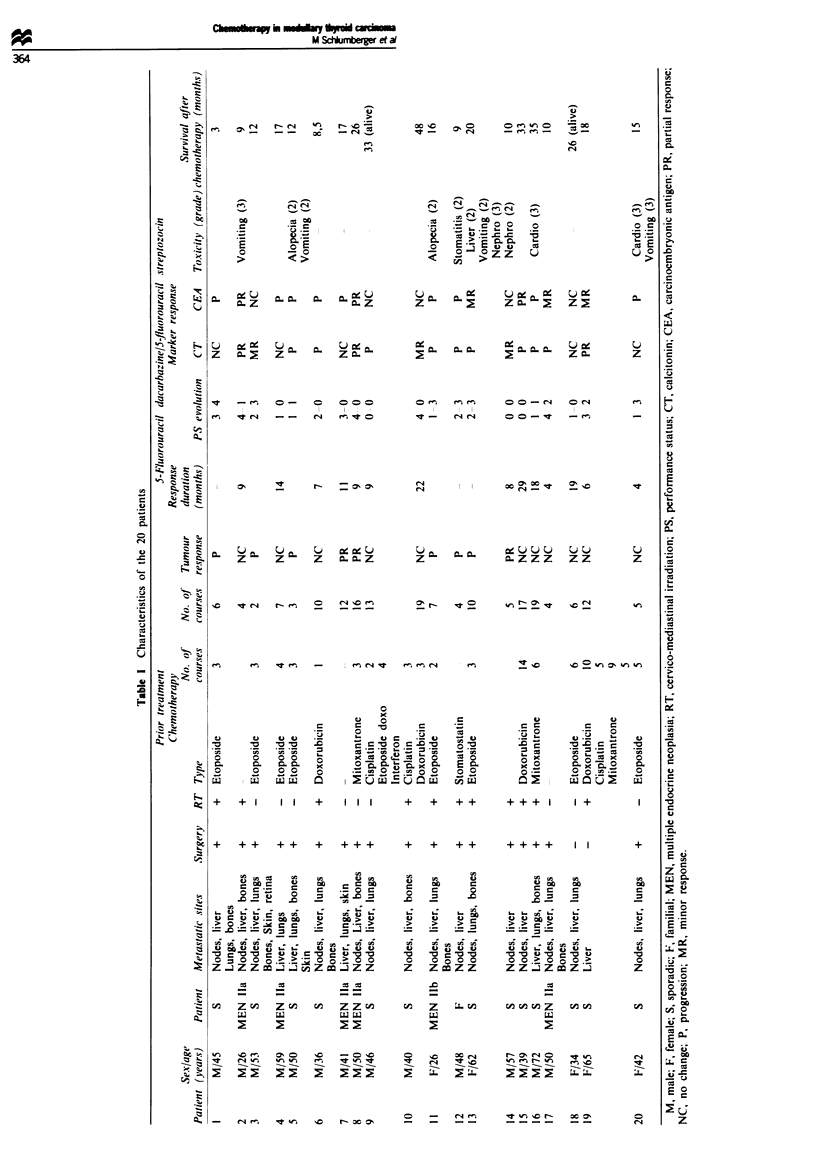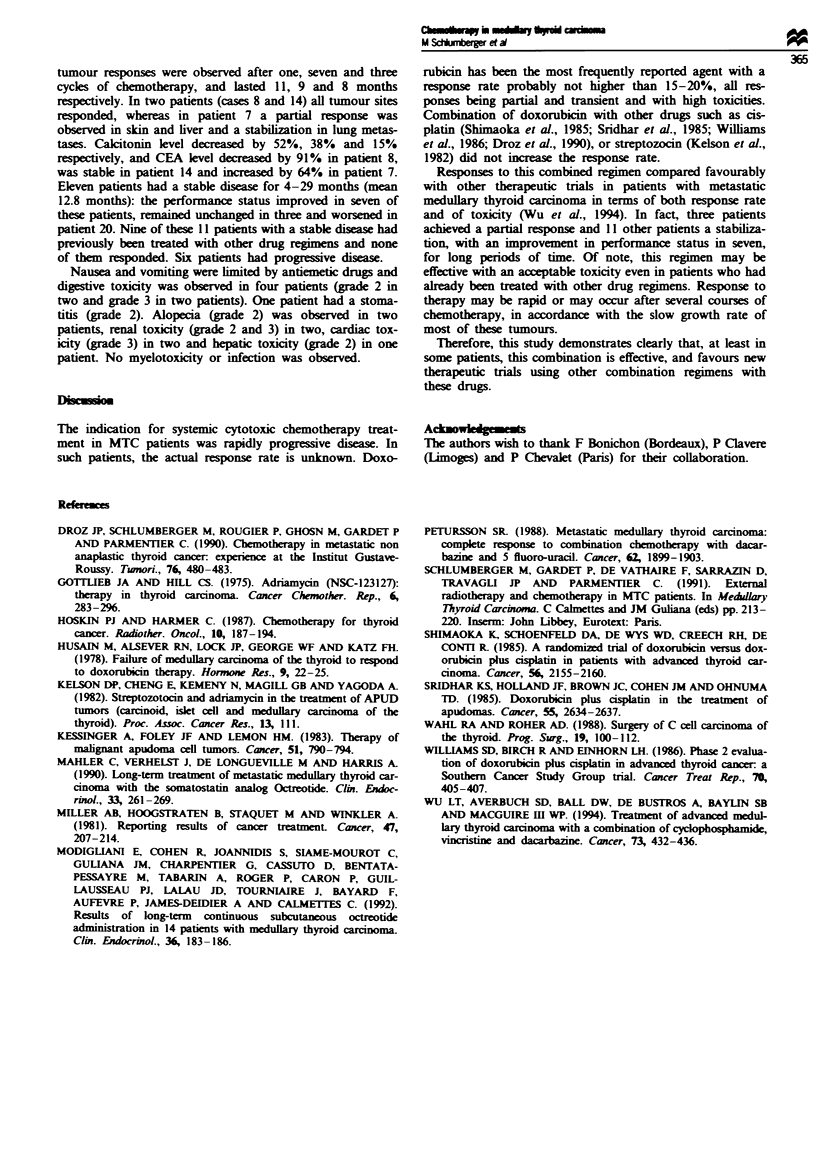# Treatment of advanced medullary thyroid cancer with an alternating combination of 5 FU-streptozocin and 5 FU-dacarbazine. The Groupe d'Etude des Tumeurs a Calcitonine (GETC).

**DOI:** 10.1038/bjc.1995.73

**Published:** 1995-02

**Authors:** M. Schlumberger, N. Abdelmoumene, M. J. Delisle, J. E. Couette

**Affiliations:** Institut Gustave-Roussy, Villejuif, France.

## Abstract

Combinations of 5-fluorouracil (5-FU) and streptozocin and 5-FU and dacarbazine were given alternately to 20 patients with metastatic medullary thyroid carcinoma. Three partial responses and 11 long-term stabilizations were observed. No unexpected toxicity occurred.


SHORT COMMUNICATION

### Treatment of advanced medullary thyroid cancer with an alternating combination of 5 FU- streptozocin and 5 FU- dacarbazine

M Schlumberger[1], N Abdelmoumene[1], MJ Delisle[2], JE Couette[3] and the Groupe d'Etude des Tumeurs á Calcitonine (GETC)

[1]*Institut Gustave-Roussy, 94805 Villejuif Cedex, France;* [2]*Institut Jean Godinot, 51000 Reims, France;* [3]*Institut François Baclesse, 14000 Caen, France.*

**Summary** Combinations of 5-fluorouracil (5-FU) and streptozocin and 5-FU and dacarbazine were given alternately to 20 patients with metastatic medullary thyroid carcinoma. Three partial responses and 11 long-term stabilizations were observed. No unexpected toxicity occurred.

**Keywords:** chemotherapy; medullary thyroid carcinoma

Medullary thyroid carcinoma (MTC) is a neuroendocrine tumour which arises from thyroid C-cells. Surgery is the main treatment when the disease is confined to the neck (Wahl and Roher, 1988). External irradiation may be indicated in patients with a detectable calcitonin level after an apparently complete surgical procedure (Schlumberger *et al.*, 1991). Chemotherapy plays no role in the early management. This disease usually follows an indolent course even at the stage of distant spread, and patients with lung or liver metastases may survive years without systemic treatment. In a minority of these metastatic patients, chemotherapy may be indicated for rapidly progressing distant metastases.

Chemotherapy trials have been limited by the scarcity of these tumours. Response rates were low with doxorubicin (Gottlieb and Hill, 1975; Husain *et al.*, 1978; Shimaoka *et al.*, 1985; Hoskin and Harmer, 1987; Droz *et al.*, 1990) and cisplatin (Hoskin and Harmer, 1987; Droz *et al.*, 1990), given either as single agents or in combination (Shimaoka *et al.*, 1985; Sridhar *et al.*, 1985; Williams *et al.*, 1986; Droz *et al.*, 1990). Furthermore, toxicities were significant. A recent trial (M Schlumberger, unpublished data) with etoposide given as a single agent did not confirm a previous report (Hoskin and Harmer, 1987), as no tumour response was observed among 16 evaluable patients. Interferon α2a (Schlumberger *et al.*, 1991) and somatostatin analogues (Mahler *et al.*, 1990; Modigliani *et al.*, 1992) did not produce any tumour response. Therefore, there is a clear need for investigation of other agents in the treatment of this disease.

In the present trial, three drugs which have been shown to be active in other neuroendocrine tumours (Kessinger *et al.*, 1983) were given, by alternating a combination of 5-fluorouracil and streptozocin with a combination of 5-fluorouracil and dacarbazine. This was further encouraged by the anecdotal report of the efficacy of 5-fluorouracil and dacarbazine in two MTC patients (Pertursson, 1988).

### Patients

From July 1986 to March 1993 20 patients (Table I) with progressive distant metastases of MTC were entered into this trial. Their mean age was 47 years (range 26-72). There were 15 males and five females. Among them, 18 had undergone a total thyroidectomy with bilateral cervical lymph node dissection, and 11 had received post-operatively external radiotherapy to the neck and mediastinum. Two patients were not operated on for diffuse distant metastases at presentation. Sixteen had been treated with one or more chemotherapeutic regimens, with drugs such as etoposide (nine patients), mitoxantrone (three patients), cisplatin (three patients) and doxorubicin (four patients), and all failed to respond.

All patients had measurable metastatic lesions that were documented by chest radiography, neck or liver ultrasonography or chest or abdominal computerised tomographic scan. The tumour markers calcitonin (CT) and carcinoembryonic antigen (CEA) were measured and followed in all patients but were not accepted as the only response criteria. The criteria used for reporting responses for measurable tumour masses were those of Miller *et al.* (1981). The tumour marker response was defined as partial for at least a 50% reduction, as minimal response between 25 and 50% reduction, no change, and progression for an increase of at least 25%.

Patients' eligibility requirements included leucocyte count greater than $4000\,\mu l^{-1}$ and platelet count greater than $150\,000\,\mu l^{-1}$, a serum creatinine of $120\,\mu mol\,l^{-1}$ or less, a total bilirubin of less than or equal to $34.2\,\mu mol\,l^{-1}$ and no chemotherapy during the preceding 3 months. Informed consent was obtained from all patients.

### Treatment

Before therapy, and also at the time of each evaluation, a medical history was taken, and the patient underwent physical examination and tumour measurements. Laboratory analysis included leucocyte count, platelet count, haemoglobin, a blood chemistry panel, ECG and assays of serum CT and CEA. Appropriate images were obtained of any indicator lesions.

Therapy was administered in the hospital. During the first course, dacarbazine was given at $200\,mg\,m^{-2}$ and 5-FU at $400\,mg\,m^{-2}$, both by intravenous injection daily for 5 days. Three weeks later, streptozocin was given at $500\,mg\,m^{-2}$ and 5-FU at $400\,mg\,m^{-2}$, both by intravenous injection daily for 5 days. At 6 weeks, this cycle was repeated. Therapy was continued until tumour progression or in case of stable disease or tumour regression, as long as there was no evidence of symptomatic or general deterioration or toxicity.

### Results

The 20 patients entered into this trial were evaluable for toxicity and for response. Each patient received an average of five (range 1-9) courses of 5-FU-dacarbazine and four (range 1-10) courses of 5-FU-streptozocin. Three partial

Correspondence: M Schlumberger

Received 21 April 1994; revised 15 August 1994; accepted 15 August 1994

tumour responses were observed after one, seven and three cycles of chemotherapy, and lasted 11, 9 and 8 months respectively. In two patients (cases 8 and 14) all tumour sites responded, whereas in patient 7 a partial response was observed in skin and liver and a stabilization in lung metastases. Calcitonin level decreased by 52%, 38% and 15% respectively, and CEA level decreased by 91% in patient 8, was stable in patient 14 and increased by 64% in patient 7. Eleven patients had a stable disease for 4–29 months (mean 12.8 months): the performance status improved in seven of these patients, remained unchanged in three and worsened in patient 20. Nine of these 11 patients with a stable disease had previously been treated with other drug regimens and none of them responded. Six patients had progressive disease.

Nausea and vomiting were limited by antiemetic drugs and digestive toxicity was observed in four patients (grade 2 in two and grade 3 in two patients). One patient had a stomatitis (grade 2). Alopecia (grade 2) was observed in two patients, renal toxicity (grade 2 and 3) in two, cardiac toxicity (grade 3) in two and hepatic toxicity (grade 2) in one patient. No myelotoxicity or infection was observed.

### Discussion

The indication for systemic cytotoxic chemotherapy treatment in MTC patients was rapidly progressive disease. In such patients, the actual response rate is unknown. Doxorubicin has been the most frequently reported agent with a response rate probably not higher than 15–20%, all responses being partial and transient and with high toxicities. Combination of doxorubicin with other drugs such as cisplatin (Shimaoka *et al.*, 1985; Sridhar *et al.*, 1985; Williams *et al.*, 1986; Droz *et al.*, 1990), or streptozocin (Kelson *et al.*, 1982) did not increase the response rate.

Responses to this combined regimen compared favourably with other therapeutic trials in patients with metastatic medullary thyroid carcinoma in terms of both response rate and of toxicity (Wu *et al.*, 1994). In fact, three patients achieved a partial response and 11 other patients a stabilization, with an improvement in performance status in seven, for long periods of time. Of note, this regimen may be effective with an acceptable toxicity even in patients who had already been treated with other drug regimens. Response to therapy may be rapid or may occur after several courses of chemotherapy, in accordance with the slow growth rate of most of these tumours.

Therefore, this study demonstrates clearly that, at least in some patients, this combination is effective, and favours new therapeutic trials using other combination regimens with these drugs.

**Acknowledgements**
The authors wish to thank F Bonichon (Bordeaux), P Clavere (Limoges) and P Chevalet (Paris) for their collaboration.

### References

DROZ JP, SCHLUMBERGER M, ROUGIER P, GHOSN M, GARDET P AND PARMENTIER C. (1990). Chemotherapy in metastatic non anaplastic thyroid cancer: experience at the Institut Gustave-Roussy. *Tumori.*, **76**, 480–483.

GOTTLIEB JA AND HILL CS. (1975). Adriamycin (NSC-123127): therapy in thyroid carcinoma. *Cancer Chemother. Rep.*, **6**, 283–296.

HOSKIN PJ AND HARMER C. (1987). Chemotherapy for thyroid cancer. *Radiother. Oncol.*, **10**, 187–194.

HUSAIN M, ALSEVER RN, LOCK JP, GEORGE WF AND KATZ FH. (1978). Failure of medullary carcinoma of the thyroid to respond to doxorubicin therapy. *Hormone Res.*, **9**, 22–25.

KELSON DP, CHENG E, KEMENY N, MAGILL GB AND YAGODA A. (1982). Streptozotocin and adriamycin in the treatment of APUD tumors (carcinoid, islet cell and medullary carcinoma of the thyroid). *Proc. Assoc. Cancer Res.*, **13**, 111.

KESSINGER A, FOLEY JF AND LEMON HM. (1983). Therapy of malignant apudoma cell tumors. *Cancer*, **51**, 790–794.

MAHLER C, VERHELST J, DE LONGUEVILLE M AND HARRIS A. (1990). Long-term treatment of metastatic medullary thyroid carcinoma with the somatostatin analog Octreotide. *Clin. Endocrinol.*, **33**, 261–269.

MILLER AB, HOOGSTRATEN B, STAQUET M AND WINKLER A. (1981). Reporting results of cancer treatment. *Cancer*, **47**, 207–214.

MODIGLIANI E, COHEN R, JOANNIDIS S, SIAME-MOUROT C, GULIANA JM, CHARPENTIER G, CASSUTO D, BENTATA-PESSAYRE M, TABARIN A, ROGER P, CARON P, GUILLAUSSEAU PJ, LALAU JD, TOURNIAIRE J, BAYARD F, AUFEVRE P, JAMES-DEIDIER A AND CALMETTES C. (1992). Results of long-term continuous subcutaneous octreotide administration in 14 patients with medullary thyroid carcinoma. *Clin. Endocrinol.*, **36**, 183–186.

PETURSSON SR. (1988). Metastatic medullary thyroid carcinoma: complete response to combination chemotherapy with dacarbazine and 5 fluoro-uracil. *Cancer*, **62**, 1899–1903.

SCHLUMBERGER M, GARDET P, DE VATHAIRE F, SARRAZIN D, TRAVAGLI JP AND PARMENTIER C. (1991). External radiotherapy and chemotherapy in MTC patients. In *Medullary Thyroid Carcinoma*. C Calmettes and JM Guliana (eds) pp. 213–220. Inserm: John Libbey, Eurotext: Paris.

SHIMAOKA K, SCHOENFELD DA, DE WYS WD, CREECH RH, DE CONTI R. (1985). A randomized trial of doxorubicin versus doxorubicin plus cisplatin in patients with advanced thyroid carcinoma. *Cancer*, **56**, 2155–2160.

SRIDHAR KS, HOLLAND JF, BROWN JC, COHEN JM AND OHNUMA TD. (1985). Doxorubicin plus cisplatin in the treatment of apudomas. *Cancer*, **55**, 2634–2637.

WAHL RA AND ROHER AD. (1988). Surgery of C cell carcinoma of the thyroid. *Prog. Surg.*, **19**, 100–112.

WILLIAMS SD, BIRCH R AND EINHORN LH. (1986). Phase 2 evaluation of doxorubicin plus cisplatin in advanced thyroid cancer: a Southern Cancer Study Group trial. *Cancer Treat Rep.*, **70**, 405–407.

WU LT, AVERBUCH SD, BALL DW, DE BUSTROS A, BAYLIN SB AND MACGUIRE III WP. (1994). Treatment of advanced medullary thyroid carcinoma with a combination of cyclophosphamide, vincristine and dacarbazine. *Cancer*, **73**, 432–436.